# Preparation of Molecularly Imprinted Electrochemical Sensors and Analysis of the Doping of Epinephrine in Equine Blood

**DOI:** 10.3390/s25010070

**Published:** 2024-12-26

**Authors:** Zhao Wang, Yanqi Li, Xiaoxue Xi, Qichao Zou, Yuexing Zhang

**Affiliations:** 1 Equine Science Research and Horse Doping Control Laboratory, Hubei Provincial Engineering Research Center of Racing Horse Detection and Application Transformation, Wuhan Business University, Wuhan 430056, China; wangzhao517@stu.hubu.edu.cn (Z.W.); x2300324512@163.com (X.X.); 2 Hubei Collaborative Innovation Center for Advanced Organic Chemical Materials, Ministry-of-Education Key Laboratory for the Synthesis and Application of Organic Functional Molecules, College of Chemistry & Chemical Engineering, Hubei University, Wuhan 430062, China; 202221106011697@stu.hubu.edu.cn; 3 Shandong Provincial Key Laboratory of Monocrystalline Silicon Semiconductor Materials and Technology, Shandong Provincial Engineering Research Center of Organic Functional Materials and Green Low-Carbon Technology, Shandong Universities Engineering Research Center of Integrated Circuits Functional Materials and Expanded Applications, College of Chemistry and Chemical Engineering, Dezhou University, Dezhou 253023, China

**Keywords:** molecular imprinting, electrochemical sensors, hydroxylated multi-walled carbon nanotubes, indium-tin sols, epinephrine

## Abstract

In this paper, a novel molecularly imprinted polymer membrane modified glassy carbon electrode for electrochemical sensors (MIP-OH-MWCNTs-GCE) for epinephrine (EP) was successfully prepared by a gel-sol method using an optimized functional monomer oligosilsesquioxane-Al_2_O_3_ sol-ITO composite sol (ITO-POSS-Al_2_O_3_). Hydroxylated multi-walled carbon nanotubes (OH-MWCNTs) were introduced during the modification of the electrodes, and the electrochemical behavior of EP on the molecularly imprinted electrochemical sensors was probed by the differential pulse velocity (DPV) method. The experimental conditions were optimized. Under the optimized conditions, the response peak current values showed a good linear relationship with the epinephrine concentration in the range of 0.0014–2.12 μM, and the detection limit was 4.656 × 10^−11^ M. The prepared molecularly imprinted electrochemical sensor was successfully applied to the detection of actual samples of horse serum with recoveries of 94.97–101.36% (RSD), which indicated that the constructed molecularly imprinted membrane electrochemical sensor has a high detection accuracy for epinephrine in horse blood, and that it has a better value for practical application.

## 1. Introduction

Molecular imprinting technology (MIT) is a technology that can mimic the process of molecular recognition in nature [1,2], such as that of antigen–antibody recognition. This is a technique in which a functional monomer and a template molecule undergo copolymerization to obtain the molecular polymerization of specific recognition sites. Molecularly imprinted polymers (MIPs) have “memory sites” that are consistent with the shape, size, and functional group orientation of the template molecule, and have good specific adsorption to the target molecule. In recent years, molecular imprinting technology has shown excellent prospects for development and application in many fields, such as chromatographic separation [3,4,5], solid-phase microextraction [6,7,8], membrane separation technology [9,10] and electrochemical detection [11,12]. EP is a class of phenylethylamine derivatives, which acts on the smooth muscle of animals to enhance cardiac contractility, promote protein synthesis, increase muscle content, accelerate fat conversion and catabolism, improve athletes’ reflexes, and improve athletes’ performance in sprinting and power performance [13]. However, epinephrine (EP) accumulates in body fluids during metabolism in the body, seriously threatening horses’ health and causing irreparable trauma. At present, the commonly used detection methods for the stimulant epinephrine (EP) in equestrian events are the urine test, blood test and hair test. Among them, the urine test is the most commonly used detection method in China, with simple sampling, high concentration, but a high probability of false positives. Hair tests make it easy to detect parent drug concentrations but tend to be costly [14]. In Europe and the United States, where equestrian event testing is mature, blood testing is often preferred, and blood testing not only solves the problem of false positives, but also provides a lower detection limit [15]. Therefore, the testing of blood samples was used in this experiment. The following assays are available for the stimulant epinephrine: gas chromatography [16,17], liquid chromatography [18,19,20,21,22] and immunoassays [23]. Gas chromatography mainly suffers from its complex operation, harsh detection conditions and the poor applicability of samples; liquid-quantity chromatography mainly suffers from low detection sensitivity and cannot be applied to routine testing; immunoassays mainly suffer from the interference by endogenous substances in the matrix. The above three methods often require the use of large precision instruments and equipment, high energy and time consumption, etc., which increases the risk of “sampling-detection”. In contrast, the electrochemical method has the advantages of being a simple device, easy operation, high sensitivity, good selectivity and low background interference. However, there are problems of poor stability and reproducibility [24,25]. Particularly, electrochemical methods of molecular imprinting in electrochemical sensor technology have a high degree of compatibility with the guiding principle of developing accurate, rapid and highly sensitive methods for the detection of doping [26].

**Figure 1:**
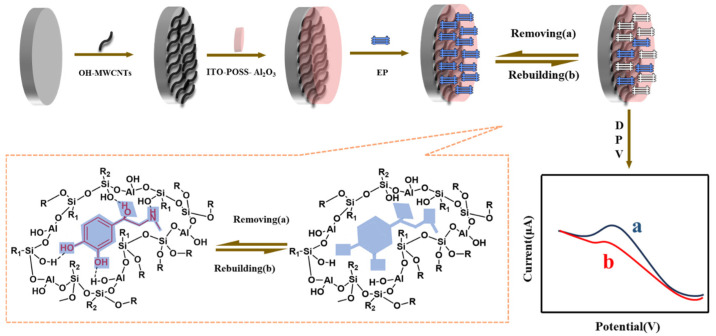
Schematic diagram of EP detection by the MIP electrochemical sensor.

In this paper, we construct a molecularly imprinted electrochemical sensor for the highly sensitive detection system of epinephrine based on the use of electrochemical technology with the advantages of being a simple equipment to operate, high sensitivity and good selectivity, taking epinephrine in equine blood as the object of study. The sensor with the best electrochemical performance was screened by a series of conditional experiments. As shown in Figure 1, first, the electrode surface is modified with OH-MWCNTs to enhance the conductivity and surface activity of the sensor. Next, the functional monomers ITO-POSS-Al_2_O_3_ and EP are optimized and fixed through intermolecular interactions (hydrogen bonding) to form a molecularly imprinted polymer (MIP). Finally, after elution with the proper solution, a molecularly imprinted electrochemical sensor with specific cavities for EP is created, which is ultimately used to detect EP in horse blood.

## 2. Materials and Methods

### 2.1. Reagents

The Epinephrine, trimethoxymethylsilane, acetic acid, ethanol anhydrous, aluminum triacetylacetone, hydroxy purified multi-walled carbon nanotubes (OH-MWCNTs), sodium dodecyl sulfate (SDS), hydrochloric acid, dopamine, isoprenaline, uric acid, procaine reagents are analytically pure and were purchased from Shanghai Macklin Biochemical Technology Co., Ltd. in China. The Al_2_O_3_ nano sol and indium tin sol (ITO) were purchased from Zhejiang ZhiTai Nano new material Co., Ltd. in China and all aqueous solutions were prepared using deionized water.

### 2.2. Instruments

Electrochemical experiments were performed on a CHI 660E workstation with a conventional three-electrode system. Material characterization was accomplished through using a field emission scanning electron microscope, atomic force microscope, Fourier transform infrared spectrometer, x-ray energy spectroscopy and QFZ-Type paint film adhesion tester (Tianjin Senrida Experimental Equipment Co., Ltd. in China), reference electrode (calomel electrode) and working electrode (platinum electrode).

### 2.3. Preparation of Electrochemical Sensors for MIPs

#### 2.3.1. Preparation of POSS

A total of 5 mol of methyl trimethoxysilane and 51.3 mL of anhydrous ethanol liquid were stirred in a 250 mL flask for 30 min. A total of 5.4 mL of distilled water, 0.1 g of glacial acetic acid and 19.3 mL of anhydrous ethanol liquid were added dropwise. After the addition was completed, the solution was heated to 50 °C for 10 h to obtain the POSS solution [27].

#### 2.3.2. Preparation of MIPs

A total of 0.01 mM of aluminum acetylacetonate was added to the POSS solution and reacted at room temperature for 0.5 h. After that, a small amount of Al_2_O_3_ solution [28] was added to the POSS gel and stirred for 2 h. Finally, ITO [29,30] was added according to a certain mass ratio (20,000:1) and reacted for 1 h to obtain an indigo-colored transparent composite sol (ITO-POSS-Al_2_O_3_). The composite sol was mixed with the template molecule EP according to a specific molar ratio at 35 °C. The composite sol and the template molecule EP (composite sol: template molecule EP = 40:1) were stirred at 35 °C for 1.5 h to obtain MIPs.

#### 2.3.3. Construction of MIP/OH-MWCNTs/GCE Sensor

A total of 7 μL (5 M) sodium dodecyl sulfate-dispersed OH-MWCNTs (10 μL) [31] solution, at a concentration of 2.5 mg/mL, was added dropwise to the surface of the polished GCE, and then cured to form the OH-MWCNTs/GCE. A total of 7 μL of MIPs were dropped on the OH-MWCNTs/GCE surface, and the surface was dried and then cured at a high temperature (80 °C); it was finally eluted with 1 M hydrochloric acid elution solution for 20 min to remove the imprinted EP, allowing us to finally obtain a MIP/OH-MWCNTs/GCE.

#### 2.3.4. Electrochemical Detection of the MIP/OH-MWCNTs/GCE Sensor

The final molecularly imprinted membrane electrochemical sensor was formed by connecting the three-electrode system of the molecularly imprinted membrane electrochemical sensor, reference electrode (calomel electrode) and working electrode (platinum electrode) to the electrochemical workstation by placing them into the culture solution. The culture solution was 5 mL of PBS buffer (pH = 7.5) with 5 μL of 1 mM epinephrine, and the elution solution was 1 mol/L hydrochloric acid. The electrochemical sensors were scanned from 0.0 V to 0.4 V using differential pulse velocity (DPV).

## 3. Results and Discussion

### 3.1. Characterization

#### 3.1.1. Characterization of Functional Monomers

**Figure 2:**
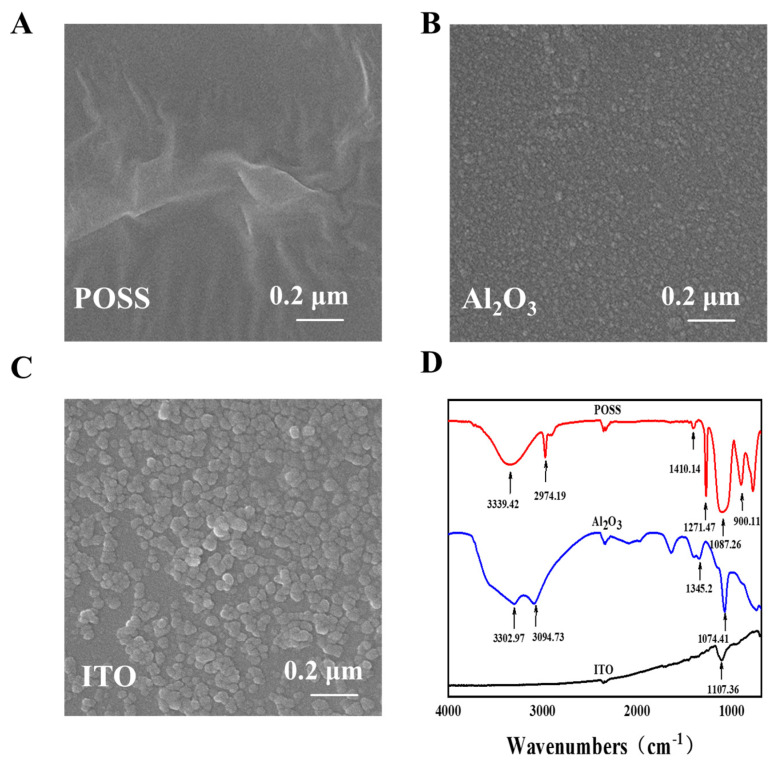
SEM images of (**A**) POSS, (**B**) Al_2_O_3_, and (**C**) ITO; (**D**) IR images of functional monomers.

The surface topographies of functional monomers were characterized by using a scanning electron microscope (SEM) (Figure 2A–C). POSS (Figure 2A) is the morphology of a wrinkled polymer film. Among them, ITO nano sol (Figure 2B) and Al_2_O_3_ nano sol (Figure 2C) are uniform spherical nanoparticles, which can increase the specific surface area of the imprinted polymer.

The chemical structures of the functional monomers were characterized by Fourier-transform infrared (FTIR) spectroscopy (Figure 2D). POSS has characteristic absorption peaks of SiO-H stretching vibration at 3393 cm^−1^, C-H_3_ stretching vibration and bending vibration at 2972 cm^−1^ and 1272 cm^−1^, Si-O-Si bending vibration at 1117.02 cm^−1^ and C-Si bending vibration at 902.78 cm^−1^. Al_2_O_3_ nano sol has an absorption peak at 3302.97 cm^−1^ due to H-O-H stretching vibration, and 3094 cm^−1^ is the stretching vibration peak of the hydroxyl group; due to the empty orbital on Al, the hydroxyl absorption peak moves like a low wavelength number and reaches the degree of deviation. The H-O-H bending vibration of water is assigned at 1640 cm^−1^, the AI-O-H bending vibration is ascribed to 1345 cm^−1^, and the AI-O stretching vibration peak is assigned at 1072 cm^−1^. Compared to the former two ITO nano sols which do not have significant absorption peaks, the oligo sesquisiloxane and Al_2_O_3_ nano sols are responsible for providing the imprinting sites for the binding with EP.

#### 3.1.2. Characterization of the MIP/OH-MWCNTs/GCE Sensor

**Figure 3:**
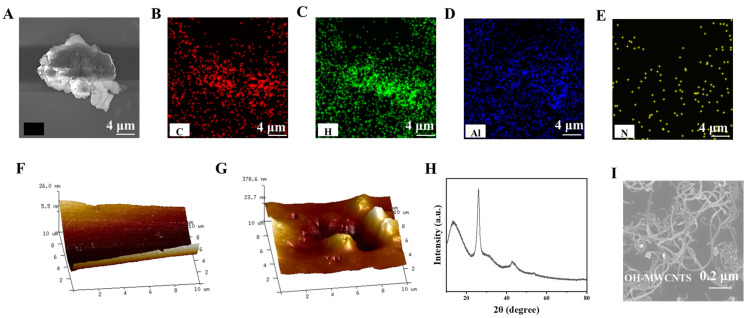
(**A**) The MIP/OH-MWCNTs/GCE sensor of EDS. EDS images of (**B**) C, (**C**) H, (**D**) Al, (**E**) N on MIP/OH-MWCNTs/GCE sensor; AFM images of the (**F**) rebuilding and (**G**) removing of MIP/OH-MWCNTs/GCE sensor, (**H**) XRD characterization and (**I**) SEM morphological characterization of OH-MWCNTs.

The elemental distribution of the MIP/OH-MWCNTs/GCE sensor was scanned by x-ray energy (EDS) spectroscopy (Figure 3A–E). EP contains imino bonds and N elements. The elemental composition of functional monomers does not have N elements. Therefore, we can look for the N element to see whether the template molecule is imprinted on the molecularly imprinted electrochemical sensor. The distribution area of N elements is shown in Figure 3E, which shows that EP was successfully imprinted on the electrochemical sensor.

The surface topography of rebuilding and removing the MIP/OH-MWCNTs/GCE sensor was characterized using atomic force microscopy (AFM) (Figure 3F,G). We used atomic force microscopy (AFM) to calculate the root-mean-square roughness (RMS) of the materials. The root-mean-square roughness of the molecularly imprinted polymer film before (Figure 3F) and after (Figure 3G) elution were 3.645 nm and 119.022 nm, respectively, which indicated that the molecularly imprinted polymer film had a sparser surface morphology after elution, which was favorable for the re-recognition of the template molecular EP by the imprinted polymer. Figure 3H,I represent the XRD characterization and SEM morphological characterization of OH-MWCNTs respectively.

#### 3.1.3. Electrochemical Characterization

Figure S4 shows the CV curves recorded before and after the elution of template molecules from the MIP film in a potassium ferricyanide solution of the same concentration in 0.1 M PBS (pH = 7.4). As illustrated, curve A represents the characteristic curve of the bare electrode in the potassium ferricyanide solution, with the oxidation peak appearing at 0.25 V and the reduction peak at 0.17 V. The symmetric appearance of the characteristic redox peaks indicates the good reversibility of potassium ferricyanide. Curve B shows that after modification with OH-MWCNTs, the redox peaks are amplified by a factor of two compared to curve A. Curve C indicates that after the successful imprinting of the MIP on the modified electrode surface, a relatively dense polymer film is formed, making it difficult for potassium ferricyanide to undergo oxidation, resulting in a weakened redox peak signal. Curve D illustrates that after the elution of the electrode, specific recognition cavities in the molecularly imprinted film allow potassium ferricyanide to undergo redox reactions at the electrode surface, leading to an enhancement of the redox peaks in the CV curve. After the MIP undergoes the re-adsorption step, the recognition cavities are filled with the template molecule EP, causing the potassium ferricyanide peak to weaken again (curve e). However, since not all eluted specific sites can be re-adsorbed, some cavities remain, and the redox peak in curve E is still relatively strong compared to curve C.

### 3.2. Optimization

#### 3.2.1. Al_2_O_3_ Sol Effect

**Figure 4:**
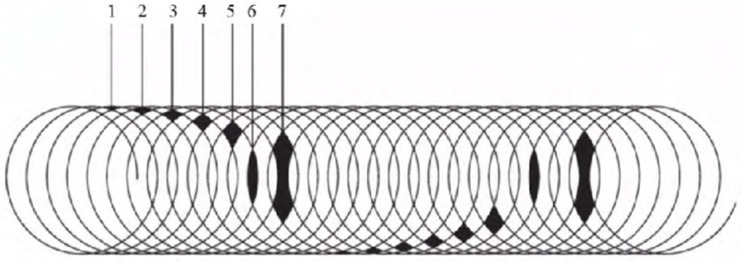
Standard chart for paint adhesion measurement.

The functional monomer was modulated according to different Al_2_O_3_ nano sol mass ratios, using the film applicator uniformly coated on the surface of the slide, at a coating thickness of 150 um, and then into at 120 °C drying for 2 h. After the slides were cooled to room temperature, the Tianjin Sen Ri Da Experimental Equipment Co., Ltd in China. QFZ-type paint adhesion test was used to test the film adhesion. According to National Standardization Administration. (2023). GB/T 1720-2023. National Standards of China. “paint film drawing circle test”, there are seven grades, from low to high, and the seventh grade is the worst. As shown in Figure 4, the sample plate to check the parts of the paint film integrity was checked. For example, if a part of the grid has more than seventy percent of the film layer intact, it is considered that the part is intact, otherwise it is considered damaged. For example, if part 1 of the paint film is intact, the adhesion level would be at 1; if part 1 of the paint film is damaged but part 2 is intact, paint adhesion would be positioned at level 2, and so on.

As can be seen from the Table 1, when the Al_2_O_3_ sol mass fraction is too low, the content is too little to be helpful. With the increase in the content, adhesion is improved; but when the Al_2_O_3_ sol mass fraction is too high, the brittleness of the polymer film is greatly enhanced, resulting in poor adhesion.

#### 3.2.2. Effect of Removing and Rebuilding Time

**Figure 5:**
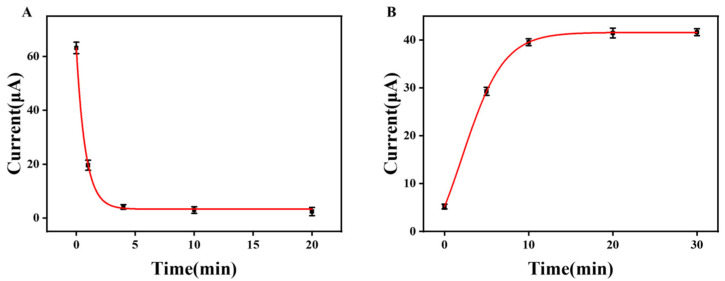
Optimization of removing (**A**) and rebuilding (**B**) times for MIP/OH-MWCNTs/GCE sensors.

To further improve the sensitivity and stability of the sensor, we optimized the molecular rebuilding and removing time. As shown in Figure 5A and Figure S2A, when 1 M of hydrochloric acid solution is used as the eluent, there is a clear absorption peak of the epinephrine molecule at the potential of 0.156 V at first, and the peak becomes weaker and weaker with the gradual increase in the removing time. The optimal removal time was 20 min. Then, the removed MIP/OH-MWCNTs/GCE sensor was inserted into the incubation solution to rebuild the EP. As shown in Figure 5B and Figure S2B, with the increase in the rebuilding time, the absorption peaks of EP were getting stronger and stronger, which indicated that the template molecules were re-imprinted on the sensor, and the value of the peak current value did not have any noticeable change when it arrived at 20 min. So, 20 min was the best time as the optimal time for rebuilding.

#### 3.2.3. Functional Monomers Solid Content Effect

**Figure 6:**
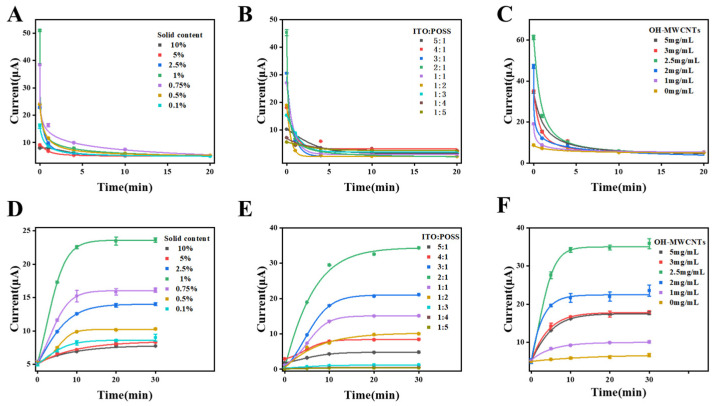
Peak current variation in sensors with different functional monomers’ solid content during removing (**A**) and rebuilding (**D**); peak current variation in sensors with different mass ratio of POSS and ITO during removing (**B**) and rebuilding (**E**); peak current variation in sensors with different modification of OH-MWCNTs during removing (**C**) and rebuilding (**F**).

The solid content of the functional monomer directly affects the number of specific recognition sites in MIPs. When polymerized with a specific amount of template molecules, if the solid content is too high, a large number of functional monomers will be distributed randomly, resulting in the formation of non-specific sites; if the solid content is too low, the distribution of functional monomers will be too small, resulting in the formation of fewer specific sites, and the polymer film will not be able to form a good cross-linking network. As shown in Figure 6A,D, the peak current reaches the maximum value when the solid content is 1% of the composite sol.

#### 3.2.4. Mass Ratio of POSS and ITO Effect

The mass ratio of POSS and ITO also has a certain performance effect. ITO and POSS-Al_2_O_3_ composite sols are close to the size of the particles, which are spherical and have a larger specific surface area to provide more blotting sites; ITO also has a certain degree of electrical conductivity and excellent durability, which increases its stability and electron transfer efficiency during electrochemical testing. As shown in Figure 6B,E, when the ratio of ITO:POSS composite sol is varied from 5:1 to 2:1, the electrical signals of the template molecules are too low, due to the high content of indium-tin sol and the low content of the composite sol, such that they cannot form a polymerization network, resulting in a reduction in the specificity of the sites for the template molecules to be bound. When the ratio of ITO:POSS composite sol goes from 1:5 to 2:1, the electrical signals of the template molecules increase, which indicate that the amount of template molecules on the electrochemical gradually becomes more extensive, which means that the number of imprinted sites of the template molecules that can be bound gradually becomes larger and larger, and the response current signals reach the maximum value when the ratio is 2:1. Therefore, the ratio of 2:1 is the best experimental condition.

#### 3.2.5. OH-MWCNTs Effect

From Figure 6C,F, the optimal concentration of OH-MWCNTs is 2.5 mg/mL. When the concentration is too low, the conductivity is not apparent; when the concentration is too high, due to the looseness of carbon nanotubes, it will lead to the poor stability of the imprinted polymer, which might fall off easily.

### 3.3. Linearity Range

The DPV method was used to test the peak current values of the molecularly imprinted membrane electrochemical sensor of the present invention after incubation in a culture medium with different concentrations of epinephrine. It was found that there was a good linear relationship between the response peak current value and the epinephrine concentration in the range of 0.0014–2.12 μM. The linear regression equation was I (μA) = 6.629 C + 1.527 (μM) (R = 99.90%), The detection limit C was 4.656 × 10^−11^ M.The sensor proposed in this experiment, as shown in Table 2, has a wider detection range and a lower detection limit compared to other EP detection methods.

### 3.4. Selectivity, Reproducibility and Stability

**Figure 7:**
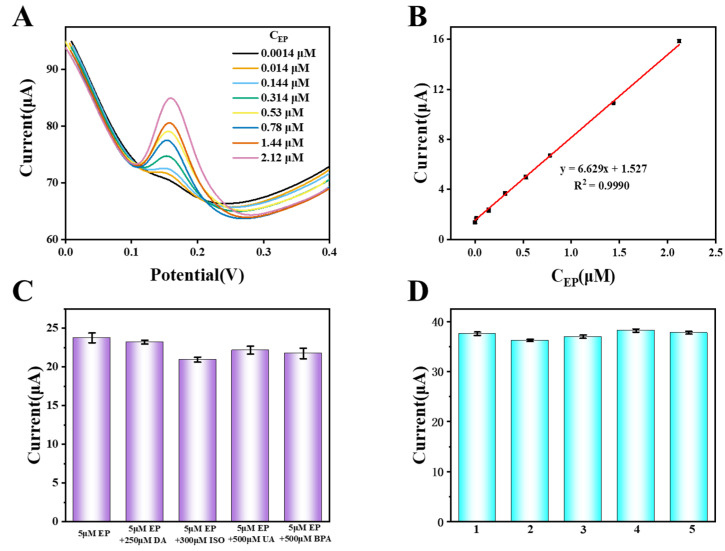
Effect of concentration on the current signals with DPV (**A**); calibration curve of EP concentrations at MIP/OH-MWCNTs/GCE sensor in pH 7.5 of PBS (**B**); specificity of MIP/OH-MWCNTs/GCE (**C**); reproducibility study of five different MIP/OH-MWCNTs/GCE sensors for 40 nM EP (**D**).

Based on the EP concentration of 5 μM in the culture medium, four other interfering molecules, namely dopamine (DA), isoprenaline (ISO), uric acid (UA), procaine (BPA), were added to the culture medium in molar multiples, and the peak current value of EP response of the MIP/OH-MWCNTs/GCE sensor developed by the present invention was tested using the DPV method in the presence of the interfering molecules. As shown in Figure 7C, compared with the presence of EP only in the culture solution, it was found that 250 μM DA, 300 μM ISO, 500 μM UA and 500 μM BPA almost did not cause any change in the peak current value of epinephrine, and there was a change in the peak current value response of only 3.4–5.0%. Thus, the MIP/OH-MWCNTs/GCE sensor constructed by the present invention still has a good selectivity for EP in the presence of interfering molecules.

DPV was used to test the response peak current value of the MIP/OH-MWCNTs/GCE sensor prepared in this study after being cultured in five culture media with the same concentration of adrenaline, and the concentration of epinephrine in the culture medium was 40 mM. As can be seen in Figure 7D, the five test results all have close epinephrine peak current signals, and the RSD value is 4.6%, indicating that the electrochemical sensor constructed by the molecular imprinted membrane has good reproducibility.

The MIP/OH-MWCNTs/GCE sensor of the present invention was first stored at 2 °C for 15 days, and then the DPV method was used to test the response peak current value of the molecularly imprinted membrane electrochemical sensor after being cultured in the culture medium, in which the concentration of epinephrine in the culture medium was 40 mM. It was found that the response peak current value of the molecularly imprinted membrane electrochemical sensor, after being stored for 15 days, was still 96.2% of the initial peak current value, and it could be proved that the molecularly imprinted membrane electrochemical sensor of the present invention could not be used in the culture medium for 15 days. Hence, this can prove the high stability of the molecularly imprinted membrane electrochemical sensor constructed in the present study.

### 3.5. Actual Sample Detection

The DPV method was used to test the response peak current values of the molecularly imprinted membrane electrochemical sensor produced by the present invention after incubation in five culture mediums with the same epinephrine concentration of 40 mM. As can be seen in Figure 7A,B, the results of the five tests have close epinephrine peak current signals, and the RSD value is 4.6%, which indicates that the molecularly imprinted membrane electrochemical sensor constructed in the present study has a good reproducibility. As can be seen from Table 3, the three samples were prepared by adding 0.1 μM, 0.05 μM, and 0.01 μM of EP to horse serum, respectively. The prepared molecularly imprinted electrochemical sensor was used for detection. The recoveries were 94.97–98.96%, and the RSD values were 1.8–4.4%, which indicated that the newly constructed molecularly imprinted membrane electrochemical sensor had a high detection accuracy for adrenaline in blood and had a good practical application value.

## 4. Conclusions

In this work, POSS, Al_2_O_3_ and ITO were used to fabricate molecular imprinted polymer films, and an MIP/OH-MWCNTs/GCE sensor was prepared by introducing OH-MWCNTs. A sensitive detection of trace stimulant epinephrine molecule in the blood of horses was achieved. SEM, AFM, EDS and FTIR methods were used to characterize the sensor construction process. By optimizing the sensor through DPV, and by improving the adhesion of the electrode surface, the problem of the stability and reproducibility of the sensor, caused by the peeling of the polymer film due to poor adhesion, is solved, the stability of the sensor is improved, and a molecular imprinting sensor with a high selectivity and high sensitivity is obtained. Under the optimized conditions, the response peak current values showed a good linear relationship with the epinephrine concentration in the range of 0.0014–2.12 μM, and the detection limit was 4.656 × 10^−11^ M. Hence, the result of this work provides a new method for the specific and sensitive detection of adrenaline agonist molecules in the blood of horses.

## Figures and Tables

**Table 1:** Effect of Al_2_O_3_ sol on the adhesion of paint film.

W (Al_2_O_3_ Solute Mass Fraction ‰)	Adhesion Level
1	4
5	5
8	3
9	1
10	7
20	7

Note: The higher the grade, the worse the adhesion.

**Table 2:** This study focuses on the preparation of a molecularly imprinted electrochemical sensor and compares its performance with other detection methods.

Detection Method	Linear Range	Detection Limit	References
HPLC	4 μM–1 μM	1 μM	[32]
UV-via	100 μM–10 μM	1 μM	[33]
DPV	1 mM–0.1 mM	50 μM	[34]
DPV	0.0014–2.12 μM	0.04656 μM	This work

**Table 3:** Detection of EP in horse blood samples.

Sample Number	EP Addition	Detection Limit	Recovery Rate	RSD
1	0.10 μM	0.0989 μM	98.96%	2.1%
2	0.05 μM	0.0506 μM	101.36%	4.4%
3	0.01 μM	0.0949 μM	94.97%	1.8%

## Data Availability

The original contributions presented in the study are included in the article, and further inquiries can be directed to the corresponding author.

## Supplementary Materials

The following supporting information can be downloaded at: https://www.mdpi.com/article/10.3390/s25010070/s1, Figure S1: Effect of Al_2_O_3_ solute mass fraction; (A) in 1‰, (B) in 5‰, (C) in 8‰, (D) in 9‰, (E) in 10‰, (F) in 20‰; Figure S2: Effect of removing (A) and rebuilding (B) time on the current signals with DPV (with a pulse amplitude of 50 mV, a pulse width of 50 ms, a pulse period of 200 ms, and a potential increment of 4 mV), pH 7.5 of PBS; Figure S3: MIP's SEM characterization graph. (a) ITO nano sol; (b) Al_2_O_3_ nano sol; (c) Oligo sesquisiloxane; (d) the surface morphology of MIP and eluted EP (e); (g) OH-MWCNTs; (h)MIP- OH-MWCNTs; Figure S4: Different electrodes in CV curves with the same concentration of potassium ferricyanide: (a) GCE (b) OH-MWCNTs/GCE (c) MIP (d) Elution (e) Re-cultivation.
