# Supplementary material for: Preparation of Molecularly Imprinted Electrochemical Sensors and Analysis of the Doping of Epinephrine in Equine Blood
Source: Sensors (Basel). 2024 Dec 26;25(1):70. doi: 10.3390/s25010070 (PMC11723366; doi:10.3390/s25010070)
Supplement: Supplementary file 1 [file sensors-25-00070-s001.zip › sensors-3358996-supplementary.pdf]

Preparation of molecularly imprinted electrochemical sensors and analysis of the doping of epinephrine in equine blood

Zhao Wang[1,#], Yanqi Li [2,#], Xiaoxue Xi[1,#], Qichao Zou[2,*], Yuexing Zhang[3,*]

[1] Wuhan Business University, Equine Science Research and Horese Doping Control Laboratory, Hubei Provincial Engineering Research Center of Racing Horse Detection and Application Transformation, Wuhan 430056, PR China

[2] Hubei Collaborative Innovation Center for Advanced Organic Chemical Materials, Ministry-of-Education Key Laboratory for the Synthesis and Application of Organic Functional Molecules & College of Chemistry & Chemical Engineering, Hubei University, Wuhan, 430062, PR China

[3]Shandong Provincial Key Laboratory of Monocrystalline Silicon Semiconductor Materials and Technology, Shandong Provincial Engineering Research Center of Organic Functional Materials and Green Low-Carbon Technology, Shandong Universities Engineering Research Center of Integrated Circuits Functional Materials and Expanded Applications, College of Chemistry and Chemical Engineering, Dezhou University, Dezhou 253023, P. R. China

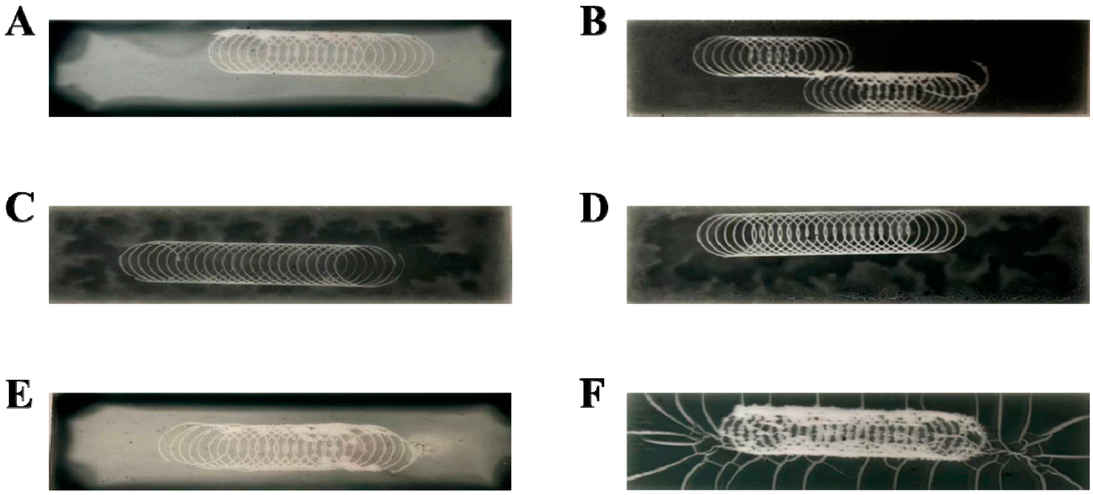

Figure S1. Effect of Al_2O_3 solute mass fraction: (A) in 1 ‰, (B) in 5 ‰, (C) in 8 ‰, (D) in 9 ‰, (E) in 10 ‰, and (F) in 20 ‰.

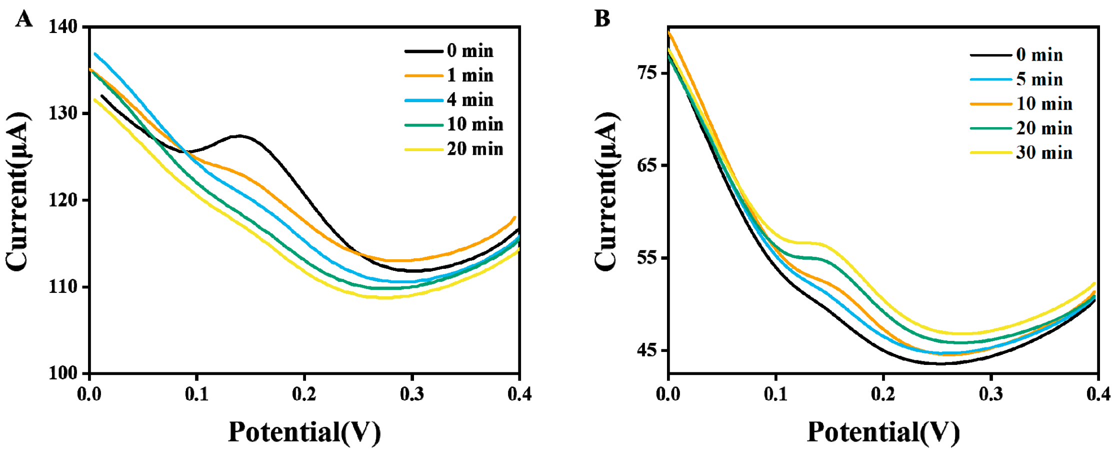

Figure S2. Effect of removing (A) and rebuilding (B) time on the current signals with DPV (with a pulse amplitude of 50 mV, pulse width of 50 ms, pulse period of 200 ms, and potential increment of 4 mV), and pH 7.5 of PBS.

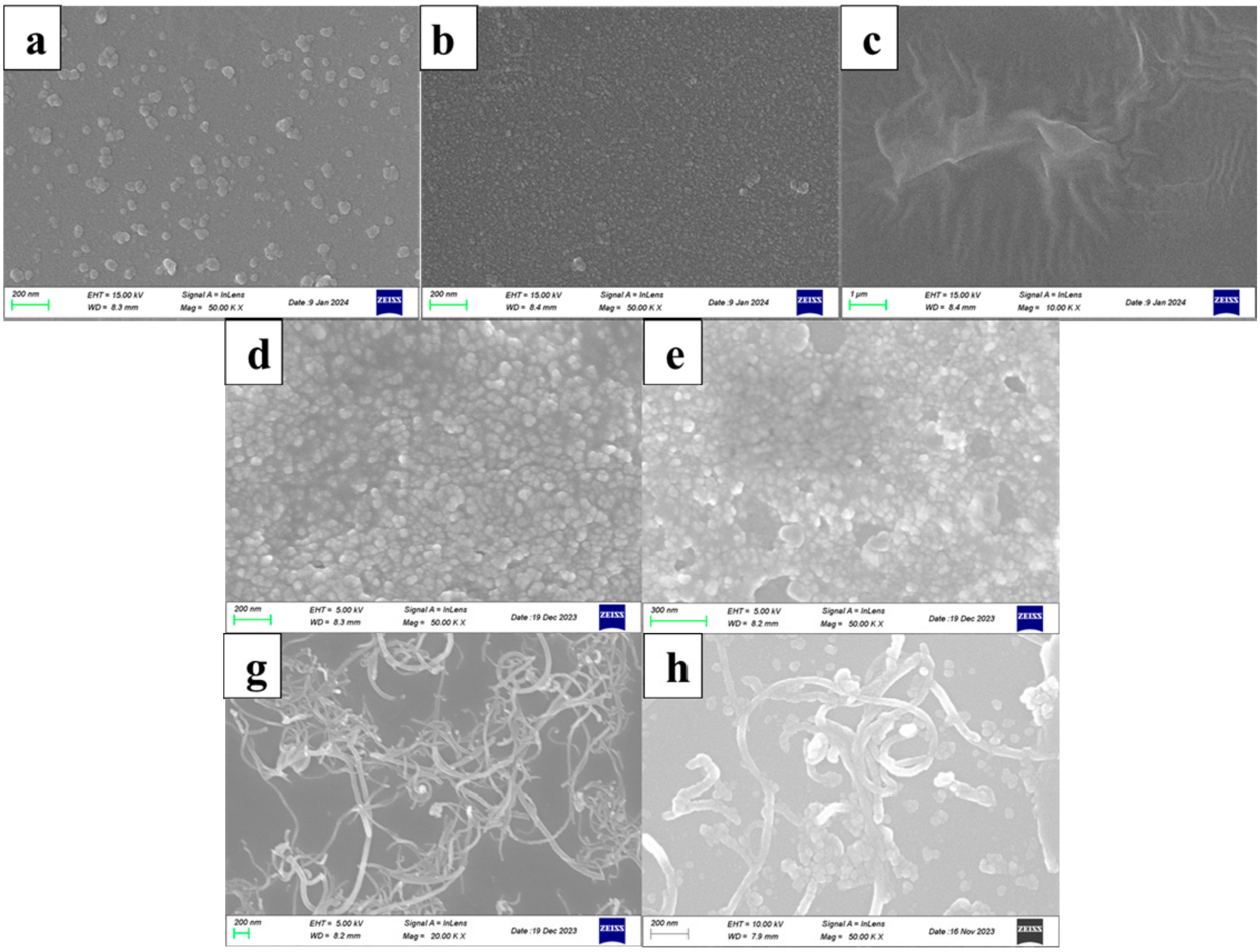

Figure S3. MIP's SEM characterization graph. (a) ITO nano sol; (b) Al2O3 nano sol; (c) Oligo sesquisiloxane; (d) the surface morphology of MIP and eluted EP (e); (g) OH-MWCNTs; (h)MIP-OH-MWCNTs.

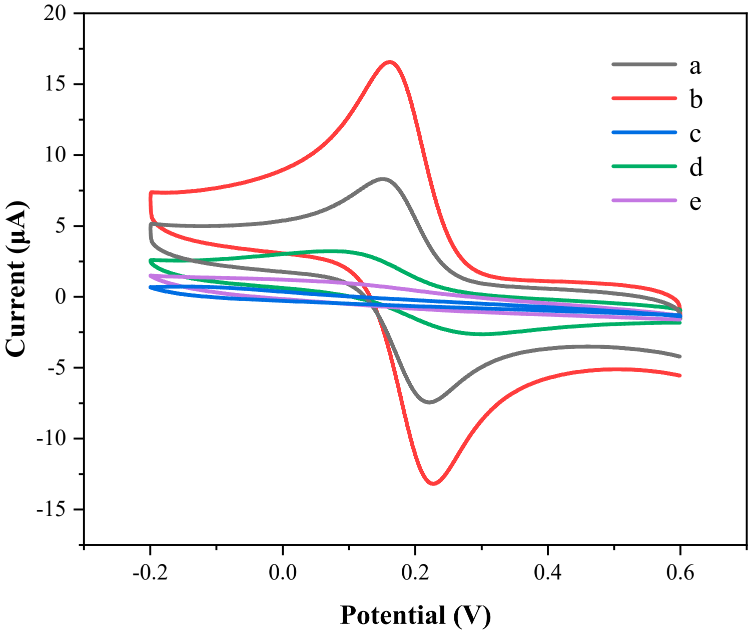

Figure S4. Different electrodes in CV curves with the same concentration of potassium ferricyanide: (a) GCE; (b) OH-MWCNTs/GCE; (c) MIP; (d) elution; and (e) re-cultivation.
